# Supplementary figures and images for: Identifying control ensembles for information processing within the cortico-basal ganglia-thalamic circuit
Source: PLoS Comput Biol. 2022 Jun 23;18(6):e1010255. doi: 10.1371/journal.pcbi.1010255 (PMC9258830; doi:10.1371/journal.pcbi.1010255)

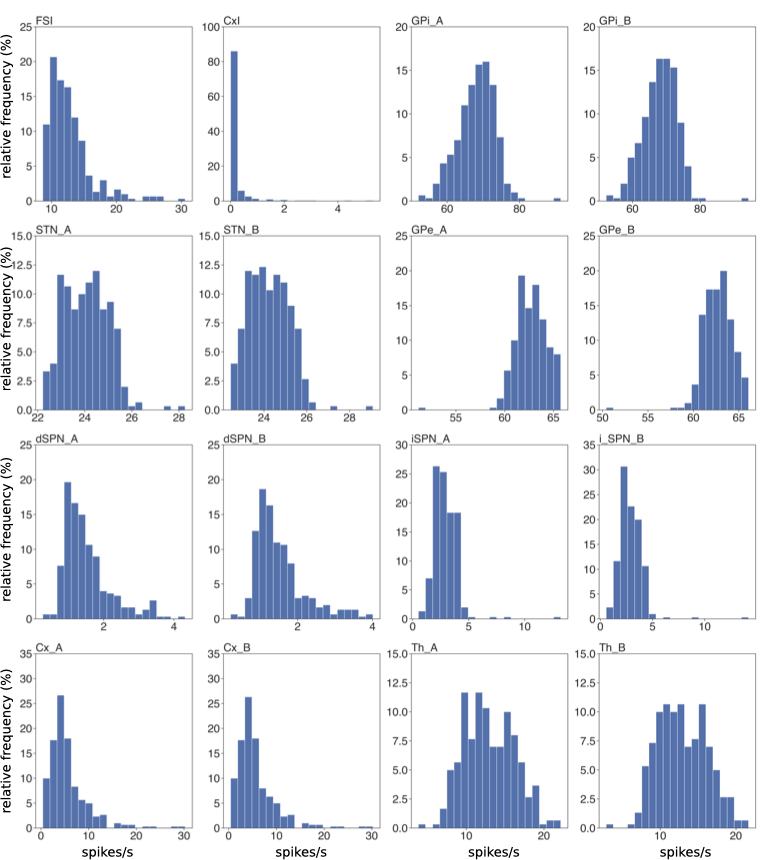

Supplement: S1 Fig — Each panel depicts, for each neuronal population, the histogram of the mean firing rates from stimulus to decision across the 300 different tuned networks. (TIFF) [file pcbi.1010255.s001.tiff]

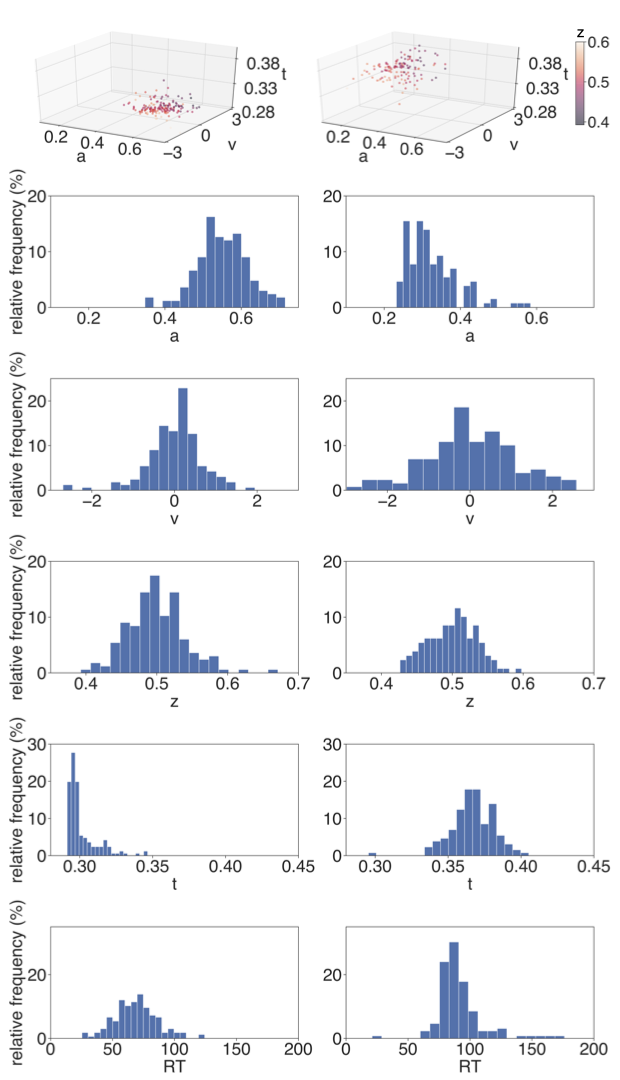

Supplement: S2 Fig — The first row shows the two main clusters of DDM parameter sets obtained after applying K-means clustering with K = 3. The third cluster consisting of just a few points with high a values is not shown here. The next four rows present the histograms of the DDM parameters a, ν, z and t, respectively, corresponding to the cluster at the top of each column. The bottom row represents the reaction time histograms for each cluster. (TIFF) [file pcbi.1010255.s002.tiff]

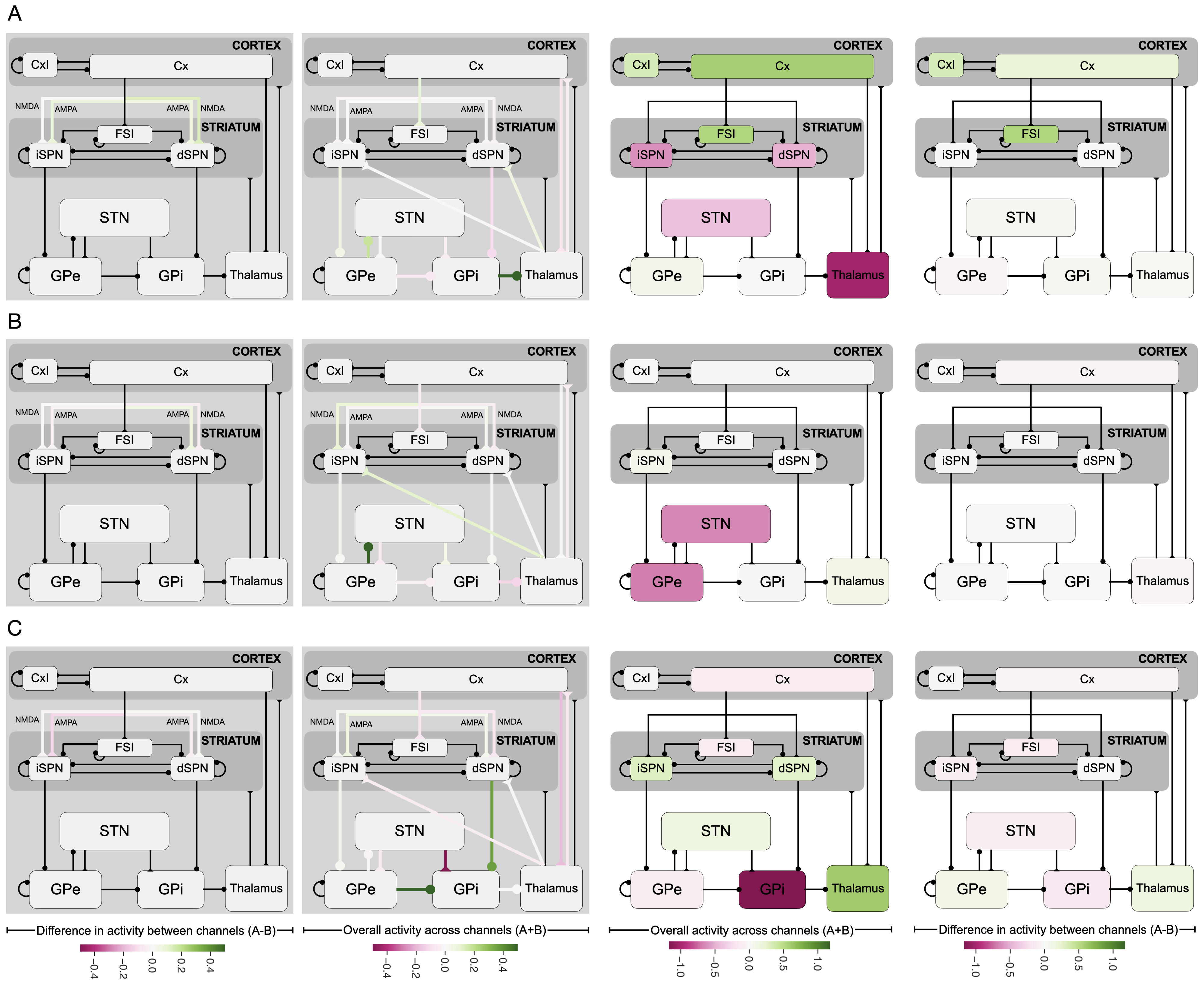

Supplement: S3 Fig — Relation between the different synaptic weights and the firing rate of each population in the CBGT network. Panels A, B and C stand for the first, second and third components of the CCA. 1st column subpanels correspond to the weight difference between channels. 2nd column subpanels correspond to the overall weights across the two channels. The loadings of the weights, shown via color-coding of synaptic pathways in the image (1st and 2nd columns) are quite weak overall; note the green pathway from GPi to thalamus in the first component (2nd column), the green pathway from GPe to STN in the second component (2nd column), and the green (magenta) pathway from GPe (STN) to GPi (2nd column). The 3rd column subpanels show the channels’ overall firing rates while those in the 4th column depict the difference in rates between the A and B channels. The color-coded loadings of the firing rates are also strong in a only a relatively small number of sites in each CCA component. (TIFF) [file pcbi.1010255.s003.tiff]

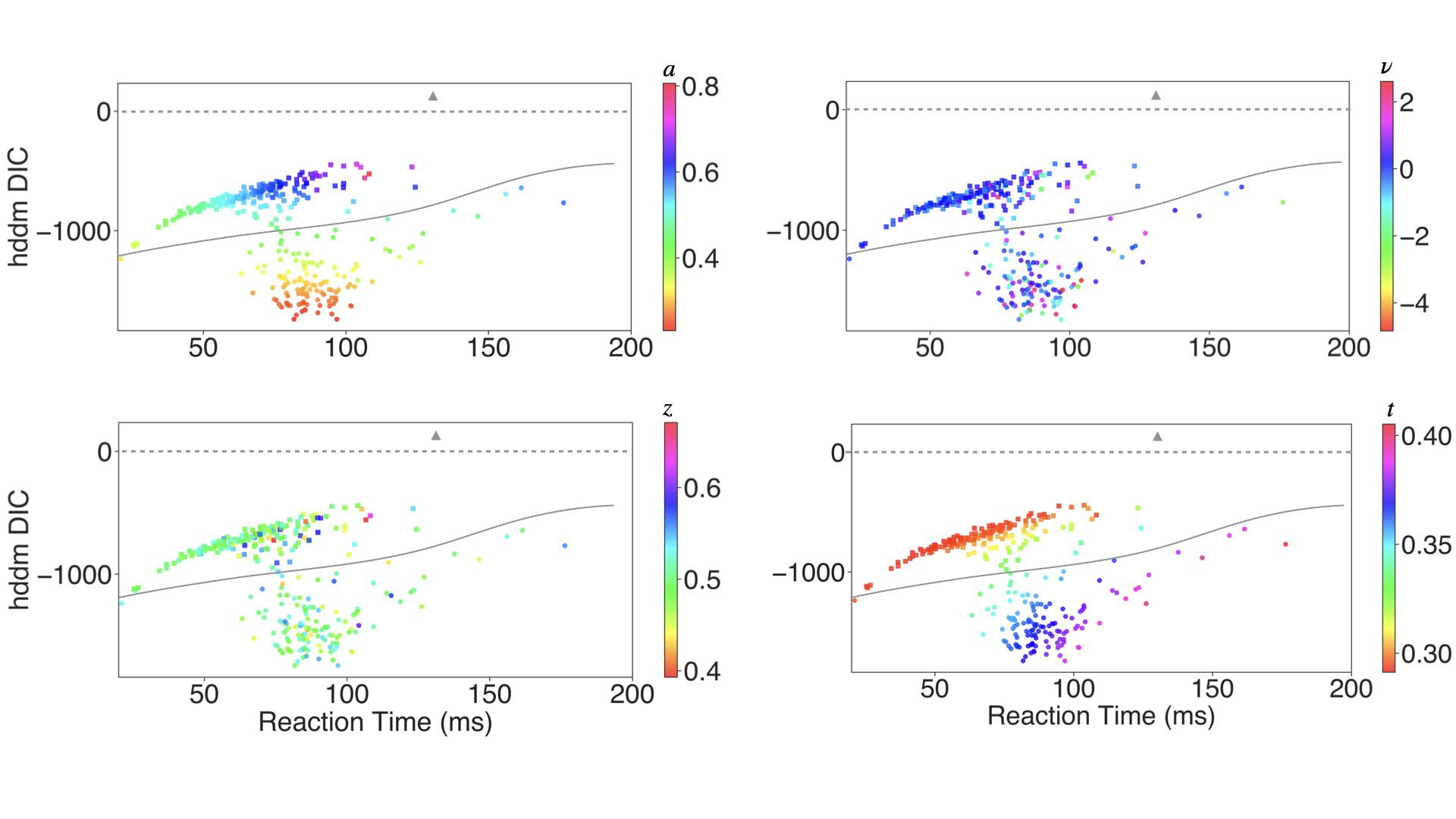

Supplement: S4 Fig — DIC values for the DDM parameters used to fit behavioral data from 300 networks tuned with different weights. The dashed horizontal line represents the DIC value equal to 0. The solid grey curve separates the two different clusters shown in S2 Fig. Data corresponding to the cluster with high t values (right panels in S2 Fig) is represented below the curve using circle markers while that corresponding to low t values (left panels in S2 Fig) is represented above the curve using square markers. Dots in the third cluster (not shown in S2 Fig) with reaction times in [20, 200] ms are plotted in grey using triangle markers. The color coding in each panel corresponds to the values of a specific DDM parameter, indicated above the color bar, in the fits. (TIFF) [file pcbi.1010255.s004.tiff]
