# Supplementary material for: Identifying control ensembles for information processing within the cortico-basal ganglia-thalamic circuit
Source: PLoS Comput Biol. 2022 Jun 23;18(6):e1010255. doi: 10.1371/journal.pcbi.1010255 (PMC9258830; doi:10.1371/journal.pcbi.1010255)
Supplement: S1 Table — From the first column to the last, we specify the receiving population, the receptor type of the external current, the frequency of the external input, the efficacy of the specific external connection, and, finally, the number of external connections projecting to the population. The time decay constant is τ = 2 ms for the AMPA receptor and τ = 5 ms for the GABA receptor. (PDF) [file pcbi.1010255.s005.pdf]

**Population**	**Receptor**	**External frequency**	**External efficacy**	**Num. of ext. connections**
CxI	AMPA	1.05	1.2	640
Cx	AMPA	2.2	2.0	800
dSPN	AMPA	1.3	4.0	800
iSPN	AMPA	1.3	4.0	800
FSI	AMPA	3.6	1.55	800
GPi	AMPA	0.8	5.9	800
GPe	GABA	2.0	2.0	2000
	AMPA	4.0	2.0	800
STN	AMPA	4.45	1.65	800
Th	AMPA	2.2	2.5	800
