# Supplementary material for: Identifying control ensembles for information processing within the cortico-basal ganglia-thalamic circuit
Source: PLoS Comput Biol. 2022 Jun 23;18(6):e1010255. doi: 10.1371/journal.pcbi.1010255 (PMC9258830; doi:10.1371/journal.pcbi.1010255)
Supplement: Attachment — Submitted filename: Vich_etal_sub2_reply-to-reviewers.pdf [file pcbi.1010255.s006.pdf]

Editor.

Your manuscript was reviewed by three reviewers. All of them have appreciated the work but have also found places where the manuscript can be improved. One of the concern is the biological relevance of the work i.e. to what extent the results of the model are applicable in a biologically plausible setting. This requires more justification for the model. Another concern is that whether bimodaltiy is a property of the model or of the fitting procedure. These concerns needs to be addressed rigorously. More details comments from the reviewers are appended below.

Reviewers.

Reviewer #1:

This paper seeks to link two levels of modelling of decision making, namely detailed biophysical models of the basal ganglia circuit and abstract diffusion model. The relationship between these models was numerically studied in the past by the Authors as well as by Ratcliff & Frank (2012), but this paper goes beyond these earlier studies and present a very comprehensive analysis. The Authors simulate the choices with the basal ganglia models with many different sets of parameters, and then estimate parameters of the diffusion model from these simulated choices. Subsequently they use nice statistical methods to describe how parameters of biophysical models are related to diffusion model parameters.

I have to admit that I have mixed feelings about this paper. On one hand, it is exceptionally clearly written, and nicely illustrated with very informative figures. All numerical analyses employ sophisticated and appropriate methods.

On the other hand, I ask myself in what ways this analysis can be useful. What can the readers gain from such analysis? Although the paper provides some motivation and experimental predictions, I think more justification for this work would be useful in the paper.

Reply: The reviewer is asking for more justification of what can be gained from our analysis. Indeed, these implications are perhaps the most useful part of the paper. Such justification is now provided in several places in the manuscript. The abstract directly describes one useful outcome of our analysis: it elucidates three neuronal control ensembles in the CBGT network, such that changes in the firing rate of each one leads to modification of a corresponding aspect of the behavior seen in a decision-making process, which can be characterized in algorithmic terms. Thus, we highlight specific regions whose perturbation (e.g., with optogenetics) should have predictable influences on behavior.

The requested justification, in more elaborated terms, is also given in the final two paragraphs of the Introduction. For example, in the penultimate paragraph, we explain that "…signals that result in changes in the activity of control ensembles will adjust factors and hence affect the behavioral outcomes that arise when using sensory signals to guide motor decisions." In the final paragraph, we describe how our analysis fills in a key missing piece: the mapping showing which neuron pools constitute the control ensembles and which decision factors each ensemble actually adjusts. (As reviewer #2 states, "To me the key insights include that specific connections and pathways in the basal ganglia can be related to DDM parameters, which can be estimated from behavioral data. While such relations have been speculated to exist before, the authors provide a model description to demonstrate this. I think this makes a lot testable predictions for studies combining electrophysiology with choice tasks.")

We do appreciate that we used some ambiguous language in some places in the abstract and introduction. Thus, we have made edits to the Abstract and Introduction to replace ambiguous phrases with clearer statements about decision-related behavior. Moreover, while the Discussion of the original manuscript treated the upward mapping between the CBGT network and DDM model (1st paragraph) separately before jumping into the behavioral interpretation (2nd paragraph), we have edited the text to provide some integration already in the first paragraph, which we hope will clarify the justification for this work.

Additionally, one can question to what extent the conclusion on the relationship of the basal ganglia model to diffusion model would generalize to relationship between biological basal ganglia to diffusion model. Although Authors used a well-developed model, presumably using a different model could give different results (e.g. Ratcliff & Frank got different conclusions with their version of the basal ganglia model). Discussion of this issue would be useful.

Reply: This is an important and interesting point, but we also note that this question about applicability of results to real biological systems applies to most or all modeling studies. We have added a new paragraph to the Discussion (at the bottom of pg. 16 and top of pg. 17) to treat this issue, where we make several points: (1) We have included a comparison with relevant aspects of the work by Ratcliff and Frank (Neural Computation, 2012), including some similarities to our current findings and also a comment on some possible reasons why we obtained some results on STN effects not observed in the previous work. (2) We have made the point, in favor of the generality of our results, that they agree with our earlier study (Dunovan et al., PLoS Comp Biology, 2019) in some ways, despite methodological differences. (3) We acknowledge that although our model is spiking-based and is tuned to match CBGT firing rates, it does omit specialized ion currents, which, in theory, could alter the conclusions of our study. (4) We express the hope that by publishing the results and predictions of our modeling study, we will provide useful ideas that will help to shape future experiments.

Despite these limitations, the manuscript provides a nice methodology for numerically linking models of different levels of abstraction, that could possibly be applied in other domains.

Minor comments:

Line 435 – It would be useful discuss the experiments on linking the neural activity in the basal ganglia with parameters of diffusion model, and to compare if the conclusions from the models match with them (e.g. Herz et al. 2017 eLife).

Reply: We do not know of many such experiments and appreciate the reference to the paper by Herz et al. We have included comments on the relation between our work and those results as well as relevant classic results of Ding and Gold (J. Neurosci, 2010) and more recent work by Yartzev and colleagues (2018). We include citations of all of these papers, in the new paragraph mentioned above (starting on line 474).

Line 582: Over what time interval, the firing rates are averaged to compute R? Is it until reaching the decision threshold, or over the whole 800ms episode?

Reply: Averaging was done from the time of stimulus presentation to the moment that thalamic activity reaches the decision threshold. We now include this information in the text (line 715).

Reviewer #2: Robert Schmidt

In their manuscript "Identifying control ensembles for information processing within the cortico-basal ganglia-thalamic circuit", Vich et al. examine how networks including the cortex, thalamus and the basal ganglia contribute to decision making. Using canonical correlation analysis they examine how changes in the synaptic weights of a network model relate to the parameters of drift diffusion models. This is important because it connects two different levels of descriptions, physiologically-grounded networks and abstract models of choice behaviour. The authors find that certain connections and pathways in the basal ganglia affect specific DDM parameters and can be interpreted in terms of responsiveness, pliancy and choice.

I think this is a very strong paper with interesting results and convincing approach. To me the key insights include that specific connections and pathways in the basal ganglia can be related to DDM parameters, which can be estimated from behavioural data. While such relations have been speculated to exist before, the authors provide a model description to demonstrate this. I think this makes a lot testable predictions for studies combining electrophysiology with choice tasks. The paper is written very well and easy to read. I have several questions, comments and thoughts that the authors can consider to further improve the manuscript.

Reply: We thank Dr. Schmidt for the complimentary comments and are pleased to learn that he finds this work interesting and useful!

1. (Figure 4) It wasn't clear to me why the data was sorted according to the order in which the trials were simulated (as these are independent anyway) and the firing rates shown don't seem to convey much information. Instead the firing rates could be grouped by the decision taken (eg a block on the left for decision=0 and one on the right for decision=1). Within each block the trials could then be sorted by RT so that a clearer relation between the firing rate and the behavioural output might be visible.

Reply: We think that this is a very good idea and different aspects can be shown if we sort the data according to the decision and/or according to the RT. We made two different versions of this plot. In the first one, as the referee suggests, we first sorted the data according to the selected action and then, for each action, we further sorted the data according to the RT, from low to high. In a second version, we only sorted the data according to the RT. Different aspects of the simulation results are evident in these representations. In the first one, we can see for some regions where firing rates in the B channel are systematically above or below those for the other channel when A is selected. This relation switches for trials where B is selected. However, after reviewing this figure, we felt that this was a subtle effect that wouldn't really add a lot to the paper. On the other hand, in the second version, by sorting only by RT, we can see how the decisions switch back and forth between A and B, which we feel that readers may easily absorb and find interesting. Because of this, we decided to replace Figure 4 with the second version of the figure, where we sort by RT, as suggested by the referee, but without separation of A and B trials.

2. (lines 184-185 & Figure 4) Did the authors consider the goodness of fit for the comparison of decision outcomes and reaction times between the CBGT and DDM models? There might also be some regions of the parameter space where the fits just don't work very well.

Reply: We have computed the deviance information criterion (DIC) value for each DDM fit. This is plotted in the new Figure S4. Apart from four sporadic cases of weight configurations that yielded a small positive DIC value (out of 300), indicating very poor fits, the DIC values are robustly negative. We repeated our subsequent analyses with these four cases omitted and saw little impact. So the results are not influenced by cases where the DDM model cannot adequately fit the simulated behavior. We have added a new comment about the success of the fits, with a reference to the new supplementary figure, in lines 207-215. Interestingly, as can be seen in Figure S4, the DIC values do reflect the cluster structure in our data; we comment on this further below in response to comment #8.

3. (lines 273-276) What was the reasoning for grouping the firing rates in this way? Obviously, taking the difference in firing rate between channels lead to interesting results as reported, but my intuition would have been to start with the firing rates averaged within each channel, separately for both decisions. Did the authors consider alternative firing rate groupings and could they add a rationale for their approach here? Related to this, for Figure 2 I was wondering why the choice-specific averages are not shown? This would also avoid the mentioned issue that the GPi decreases are barely visible.

Reply: Thank you for this comment. We have changed Figure 2 to include data averaged only over those trials where action A was selected. Now it can be appreciated that the drop of activity in the GPi precedes the increase of activity in the thalamus for the A channel just before the decision time. The title of the figure and the text related to it have been modified appropriately.

As for the use of sums and differences of activity across channels within each population, we have added a brief comment at the start of the "**Firing rates to decision parameters"** subsection to mention that these types of measures proved useful in our previous, related work (Dunovan et al., PLoS Comp. Biol., 2019). An advantage of this grouping is that it lets us observe patterns that occur across trials regardless of which decision was made; we also briefly mention this point in the revised text.

5. Throughout the manuscript the authors examine the scenario with the evidence for A and B being the same. What is the reason for looking at that scenario exclusively, and not consider cases where the decision is biased?

Reply: This work was designed to establish a clear mapping between CBGT population firing rates and decision factors. Indeed, it is only with equivalent sensory signals that we can discern relations between CBGT network dynamics and behavior, as introducing a bias between the choices driven by the sensory signals would alter simulated decision outcomes and DDM fits in a way that would confound and complicate the derivation of the mapping (though this question is a focus of our future modeling projects). For example, if we were to introduce bias in the sensory signals, how many different levels of bias would we need? Should we include all results under all levels of bias in one mapping analysis? What if the tuning effects of control ensembles depend nonlinearly on bias in evidence? By avoiding these complications, we were able to produce and describe what we hope are a clear set of methods, results, and predictions. In future work, we plan to build on our baseline findings by allowing for asymmetric evidence and corticostriatal plasticity. We have revised the text in the Discussion, lines 511-513, in a way that includes this point.

6. In the model analysis, changes in the weights are considered. However, the number of neurons in each population and their connection probabilities also have a large effect on the network activity. What were those parameters based on, e.g. taken from a previous model and/or based on experimental studies (e.g. in Table 1 and 3)? This might e.g. affect the GPe/STN interaction and whether weight changes manifest as a net excitation or inhibition in GPi.

Reply: We apologize for not providing more information about these aspects of the model. We have revised the relevant text to include more details about this and other related modeling choices. Following earlier work using this model (Wei et al., J. Neurosci, 2015; Dunovan et al., PLoS Comp Biol., 2019), we use a relatively large collection of GPe neurons to allow the model to include the sparse connectivity of projections from striatum to GPe, and then we use the same number of STN neurons as GPe neurons because connections from GPe to STN are sparse, and with fewer STN neurons in the model the indirect pathway loses efficacy.

7. (line 241) It is mentioned that the 2nd CCA was to address the 'primary hypothesis of interest'. I don't think it is stated anywhere what that hypothesis is.

Reply: Thanks for raising this good point. We have revised the text to state the hypothesis clearly up front.

8. In Figure 5 the authors show that in their random sampling of network weight parameters two main clusters emerge. As far as I understand it, the CCA analyses are also based on the random sampling of 300 network configurations. I was wondering to what degree the overall results, ie the mapping between R and P depends on the sampling. For example, network configurations belonging to the 'High a and low t' cluster might be systematically different in their activity patterns than network configurations belonging to the other cluster. In the CCA analysis network configurations from both clustered are included by the random sampling. Could this affect the results in terms of the found loadings? Would the loadings be similar if the CCA analysis was performed for network configurations from each cluster separately?

Reply: Our fitting process produces two clusters of DDM parameters. The lower DIC cluster is (low a, high t) and the higher DIC cluster is (high a, low t). In the revision process, we repeated the stochastic fitting process 50 times for a variety of network configurations for which the original fit gave DDM parameters in the (low a, high t) cluster and another set of network configurations for which the original fit gave DDM parameters in the (high a, low t) cluster. We found that there was complete consistency in the new fits; that is, all new fits belonged to the same cluster in DDM parameter space as did the original fit. We have added new text in lines 250-254 to present these confirmatory results.

These clusters correspond to different RT distributions (e.g. see the new Supplemental Figures S2 and S4) and hence are needed to allow the DDM model to capture the full range of CBGT behavioral data. Thus, we conclude that both clusters should be included in our CCA analysis to fully represent the mappings that we have uncovered, as is now explained in lines 254-262.

9. (line 367): the authors mention that the CCA applied directly to W->P did not work as well. While reading your manuscript I was also wondering at an earlier section about why you did not just use W->P directly. What are possible reasons for why that approach does not work well? Above (l. 447) you had motivated your approach by firing rates being experimentally more accessible, but then here (l 367) it just sounds like that approach did not work well, so why is that?

Reply: This is another good point. The issue here is that network firing rates are what directly impact simulated behavior, and synaptic weight changes lead to complicated effects on firing rates that are not well revealed by the CCA between W and R, which is dominated by a few specific effects (Supp. Fig. S3). We have edited lines 406-421 to clarify this point.

Minor points / suggestions:

Figure 5: I would normalise the histograms (eg to %) instead of raw frequency so that it is easier to interpret independent of the number of trials run in the simulations. In panels C and E I was wondering whether the data partly clustered at the lower end of the allowed parameter range? Or were a and t allowed to fall below 0.25 and ~0.29 and the high frequency there is not due to a lower cap?

Reply: Thanks for the good suggestion. We have implemented this normalization. At the same time, we have also normalized the histograms in Figures S1 and S2. For the second question, no bounds were placed on these parameters but rather the distribution emerged from the fits. We added a sentence to mention this point on lines 235-237 of the revised manuscript.

Figure S3: I struggled to understand the 3rd and 4th column - what is the interpretation for the color in a given region?

Reply: The color-coding of this figure is indicated at the bottom of each column and is equivalent to those in Figure 8. In each row, the colors in the first two columns represent the loadings of synaptic weights and the colors in the final two columns represent the loadings of the firing rates. We believe that the reviewer's confusion may have related to the fact that the color codings in the first two columns are very easy to miss, because they appear on the synaptic pathways rather than on specific regions and only a small number of pathways are loaded significantly in each component; thus, we have added text to draw attention to the colorings in the first two columns in the caption, and have made some other minor, clarifying revisions there. We believe that these updates, together with the text in the **"Weights to firing rates"** part of the Results section, should prevent reader confusion.

Figure 6: As the components can only be integer numbers, I would not show the x-labels & tick marks for fractions (e.g. "2.5") for clarity. I was wondering initially why only up to 4 components are shown in panel B, but I think the reason is that the 2nd CCA is limited by the number of components chosen for the 1st CCA? If so, this might be helpful to mention somewhere.

Reply: The x-labels of Figure 6 are now changed according to this suggestion. The referee is also right about the reason why we consider only up to 4 components for the 2nd CCA. This point is explained in the Methods sections, subsection 4.4.1 (line 733), where we specify that the number of components is selected to be less than or equal to the minimum of the dimensions of the two data sets involved in the CCA. We have added this comment to the caption for Figure 6.

line 289: This is rather subjective, but the term 'pliancy' didn't seem very intuitive to me for describing the 2nd factor. Doesn't it somewhat correspond to what has also been referred to as 'urgency' in the work of Paul Cisek and others, e.g. here https://doi.org/10.1523/JNEUROSCI.1844-09.2009 ?

Reply: We agree that "pliancy" is not an obvious word choice, but careful thought shows that it is the right term for the effects that we are describing. Urgency would be something distinct from what we are after; urgency would combine earlier onset time and lower boundary height, representing a situation with pressure to act fast, whereas our factor two is more about the boundary height alone, representing control of the standard of evidence

needed in order to commit to a decision or the willingness to be convinced. These concepts are what the word "pliancy" captures.

For the understanding of Figure 7 and 8 it might be good to clarify somewhere that they actually contain the same data and are two different visualizations of the loadings (if I understood it correctly…)

Reply: We have added new text in lines 330-331 to provide this linkage between the two figures.

lines 150-252: Not clear which studies and data sets you are referring to, please add some references.

Reply: It's not clear to us where in this section the addition of references would be helpful, other than references about CBGT population firing rates. These references already appear in Table 2, so we have simply added some clarifying text before our reference to Table 2 in line 151 of the revised text.

line 305: unclear, in what sense is the cortical impact more 'subtle'?

Reply: Thank you for catching this erroneous text, which was left over from an earlier version of the manuscript and which we have deleted.

line 377: I would suggest to phrase this a bit more cautiously, as some people (like me) would also consider e.g. arkypallidal and prototypical neurons to comprise 'major basal ganglia populations'.

Reply: We have replaced the word "major" with the phrase "classically recognized" in this text, which is now in line 459.

line 386: Dopamine effects are mentioned here. I think even without considering other scenarios or explicitly modelling dopamine neuromodulation, you might already be able to make some predictions on the effect of dopamine, based on the simplified view that dopamine would overall excite D1 SPNs and inhibit D2 SPNs. In particular, the dSPN effect on responsiveness would indicate that D1 receptor activation reduces t and a; D2 receptor activation reduce a but increase t; and dopamine release in general would increase v and decrease z. As there are already many studies on the effects of dopamine on choice behaviour you might draw some interesting connections there.

Reply: This is an interesting idea. We did an exploratory analysis looking at how variability in the different corticostriatal synapses associates with each DDM parameter. Maybe not so surprisingly, there was no clear relationship between individual synaptic weights of the various D1 and D2 receptors and individual DDM parameters. In retrospect, this makes sense given that we explicitly exclude dopaminergic influences in our model simulations here, although this is an interesting area of follow up research that we are currently exploring.

lines 27-29: "D1-expressing spiny projection neurons (SPNs) in the striatum, unleashes GABAergic signals that can suppress activity in the CBGT motor output nucleus (internal segment of the globus pallidus, GPi, in primates or substantia nigra pars reticulata, SNr, in rodents)".

I would rephrase this statement, as it seems to suggest that rodent SNr is the equivalent of primate GPi, or that primates don't have an SNr.

Reply: We have changed the wording in lines 28-30 to avoid this misunderstanding.

Reviewer #3: Mark Humphries

This manuscript's goal is to understand more deeply the roles of of cortico-BG-thalamic (CBGT) components in decision-making tasks, by continuing a great line of work mapping the complex CBGT circuit to tractable, simpler models. The approach taken here is to set up a complex spiking-neuron CBGT model whose firing rates fall within reasonable ranges; fit a DDM model to reaction times and choices made by the model in response to identical inputs for two choices; repeat for 300 sampled weight sets; then use CCA to study how changes in firing rates correlate with changes in DDM parameters. The CCA analysis revealed a mapping between three key elements of decision making the activity shared among specific components of the CBGT circuit. The manuscript clearly reports a substantial body of work, with a clear goal, some solid, interesting results, and a novel use of CCA (to me, at least).

Main suggestion below are towards further refining the paper to make elements clearer for the reader, and head off some potentially nagging concerns they may have:

- Introduction (lines 46-62): whole paragraph on action selection vs vigor, followed by claim that CBGT's proposed roles in action selection and vigour can be reconciled by understanding how the pathways within the CBGT process information. This is not referenced again in the paper, and is unclear how the present work address the issue. Seems tangential to the main aim of getting a handle on how CBGT circuit maps to decision making

Reply: We apologize for the lack of clarity on our part. The making of a decision, like any other behavior, can be done with various levels of vigor. DDM model parameters can be tuned in ways that prioritize accuracy or speed, the decision-making analogue of vigor. Of the three decision factors that our work identifies, two of them, responsiveness and pliancy, relate to the briskness with which decisions are made. This connection to vigor is therefore, we feel, present throughout our presentation of our results. Nonetheless, we have made revisions in multiple places in the Introduction and Discussion (including a new paragraph on lines 422-441 describing the relationship between vigor and decision making) to reference this point explicitly.

- Results:

- A major result is that the DDM fits fall into two clusters, suggesting two main parameterisations of decision-making in this CBGT model, based only on variation in boundary height and onset (lines 218-222). How do we know this bimodality is a real property of the CBGT model and not a consequence of the DDM fitting procedure? One could imagine that the DDM fitting procedure has two local minima, corresponding to each of these modes, such that re-running the fitting would put a given CBGT model in a different cluster.

Reply: As stated in our response to comment 8 of reviewer #2, we repeated the stochastic fitting process 50 times for a variety of network configurations for which the original fit gave DDM parameters in the (low a, high t) cluster and another set of network configurations for which the original fit gave DDM parameters in the (high a, low t) cluster. We found that there was complete consistency in the new fits; that is, all new fits belonged to the same cluster in DDM parameter space as did the original fit. We have added new text in lines 250-255 to present these confirmatory results.

- Given that there are two clusters of fits, how do we then interpret the CCA analysis of (R->P), when CCA measures linear correlation but the DDM parameters are discontinuous?

Reply: Methodologically, there is no problem with the CCA used here. In any CCA analysis, the data points are necessarily discrete, because only a finite number of simulations can be performed. The CCA is designed to work with discrete data and to identify directions along which correlation is maximal between both inputs and a multivariate output. Although the CCA could be performed separately on each cluster rather than on the whole data set, our additional simulations (see response above) confirm that the clusters correspond to different RT distributions (e.g. see the new Supplemental Figure S2) and hence are needed to allow the DDM model to capture the full range of CBGT behavioral data. Thus, we conclude that both clusters should be included in our CCA analysis to fully represent the mappings that we have uncovered, as is now explained in lines 255-262.

- how do we read the relationship between the canonical loading matrices in Figure 7A and Figure 7B? This relationship underpins all the main conclusions on the mapping from CBGT models (Fig 7A) to the decision making process (Fig 7B) in lines 282 onwards, but the connection between the two matrices is never made explicit. For example, what are the canonical loadings measuring (i.e. why do we care about the correlation between the canonical vectors and the original data?) And given that we are comparing between pairs of

like-numbered columns in Figs 7A and 7B, perhaps explain how we should make that comparison - presumably a canonical loading on one axis (e.g u) imply a strong relationship on the other (e.g. v) for a given mode?

Reply: We have added new sentences to the text introducing Figure 7 to clear up this confusion, which read as follows, “The loadings of each firing rate component can be interpreted as coefficients in a linear combination of the indicated firing rate measures, representing a vector $\mathbf{u}$ in the corresponding 16-dimensional space, and the loadings of each corresponding DDM parameter component as coefficients in the linear combination of these quantities that represents the vector $\mathbf{v}$ in DDM parameter space most strongly correlated with $\mathbf{u}$. The three $\mathbf{(u,v)}$ pairs identified represent the most strongly correlated vector pairs identified from these two spaces.”

- Discussion could be improved in places:

- a W->P mapping is mentioned, but is not in the Results

Reply: We apologize for introducing confusion here, as was also noted by Reviewer 2. We performed a W->P mapping and, like the W->R mapping, it did not reveal very helpful results or explain much of the variance in the data. We prefer not to mention this in the Results as it would distract from the useful results of the analysis that we describe. We have revised the relevant text in the Discussion to clarify this point and have included the phrase “(data not shown)” specifically about this mapping.

- perhaps join the dots for the reader: what do we learn from W->R and R->P mappings together? I.e. what changes in synaptic weights would alter DDM models? Or do we need the W->R mapping at all?

Reply: The most informative analysis in our paper is the R->P mapping. However, to our thinking, a natural starting point was to consider the W->P mapping, because the synaptic weights are the quantities that are most readily modifiable biologically. We performed this mapping and found it was not informative. The results of the W->R mapping help to explain why: this mapping is dominated by clear-cut effects of a small number of pathways, while other pathway effects are presumably diluted by the feedback loops in the CBGT circuit. We feel that these observations, which emerged from our analysis, will be useful to readers, who would otherwise wonder about the emphasis on the R->P results. Putting our W->R and R->P results together does not give a clear picture of what changes in synaptic weights would alter decision policies, unfortunately; in fact, an interesting interpretation of our work is that we do not expect there to be simple CBGT synaptic weight modulation mechanisms that directly yield decision policy updates.

We thank the reviewer for prompting us to think through these points. We have added text to the discussion (lines 409-421) to address them.

- predictions: there seem to be some quite deep predictions of how causal interventions in the CBGT loop would change decision making parameters in experiments - I'd encourage a clear explicit account here, to aid the interested experimentalist

Reply: We adapt part of our response to the related first comment of Reviewer #1: A collection of major predictions from our work relate to the three neuronal control ensembles that it identifies in the CBGT network, such that changes in the firing rates associated with populations in each ensemble are predicted to lead to modification of a corresponding aspect of the behavior seen in a decision-making process, which can be characterized in algorithmic terms. As reviewer #2 states, “To me the key insights include that specific connections and pathways in the basal ganglia can be related to DDM parameters, which can be estimated from behavioral data. While such relations have been speculated to exist before, the authors provide a model description to demonstrate this. I think this makes a lot testable predictions for studies combining electrophysiology with choice tasks.”

While the Discussion of the original manuscript treated the upward mapping between the CBGT network and DDM model (1st paragraph) separately before jumping into the behavioral interpretation (2nd paragraph), we have edited the text to provide some integration already in the first paragraph, which we hope will clarify the predictions associated with this work. Moreover, we have revised the start of the fourth paragraph of the Discussion to make some additional explicit statements about predictions.

Minor suggestions/queries for text and figures:

- Abstract: it is not clear what "responsiveness" and "pliancy" are until deep into the paper

Reply: In the abstract, we have added a phrase associated with the label for each decision factor to clarify what each factor represents.

- Figure 1: legend says three decision making trials, but there seem to be four pairs of vertical black/green lines? Would also be useful to plot the decision threshold (30 Hz) on the thalamus output panel

Reply: We agree with the referee that there were actually 4 decisions. We have changed the figure to remove the fourth action since it was a little bit hidden at the end of the simulation. In addition, we have added the decision threshold to Figure 1 (Th subpanel) and mention it in its caption.

- line 144-145 "varying... specific connections" - would help reader here to have some idea of which connections are being sampled, to summarise Table 3

Reply: We have changed the text in these lines to “varying the synaptic conductances of many of the connections…” but do not want to interrupt the flow of the manuscript by providing more detail here (note that Table 3 is already referenced). In the Methods, in subsection 4.2 where we reference Table 3, we have added text to provide the summary and some rationale for our choices, as the reviewer suggests (lines 661-665).

- line 153 "each permuted network" - unclear what was permuted? "Sampled" meant here?

Reply: You are correct; we have replaced “permuted” with “sampled” here and in one other instance where it occurred.

- Figure 3: perhaps draw horizontal line at 0, to make clear the sign of each coefficient (and mapping to synapse's sign)

Reply: We have revised the figure to include this line.

- lines 169-170 and Figure 3: claim that many populations are insensitive to variation of synaptic weights. As a linear model was used here, is it more accurate to say the firing rates are not monotonic function of the variation in weights?

Reply: We have changed the text to state that many firing rates do not exhibit a systematic linear variation under changes in weights.

- lines 181-185, "The behaviour..." onwards: perhaps delete as this was rather confusing, and then explained in full anyway from lines 197 onwards.

Reply: We have deleted the earlier, redundant lines and slightly revised the later lines for completeness.

- lines 188-194: perhaps explicitly give the RTs predicted by the model, which would seem to be about 150-350 ms given standard "execution time", then give citations to papers reporting these RTs

Reply: The RT’s predicted by the model are shown in Figure 5. We now include additional citations to support the timing of our model’s selections. It is important to point out that the “reaction times” of our model most closely reflect the response times of the cortex making the decision (i.e., before motor planning and execution begins). We now make this clear in this section, with additional citations to justify that the “reaction times” of our model match onset of cortical activity to initiate a decision in cortical planning populations.

- Figure 4: implies all model outputs are used to fit DDM model. Perhaps plot intermediate step of choice and RT distributions, and show DDM fitted from these

Reply: We have not included a new plot because we feel that this would be redundant with the similar plots in Figs. 5A,B.

- lines 207-210: suggestion here that drift rate and bias are normally distributed - true, or just unimodal?

Reply: We have performed the Saphiro-Wilk test in Python and seen that they are just unimodal, so we have changed the wording here.

- Figure 5: could not see the z parameter variation along the drift axis in this 3D plot. Perhaps give two 2D colour-coded plots to show main directions of change referred to in the text.

Reply: After plotting the two 2D color-coded plots to show a-t-z and v-t-z relations, we found that results using these 2D plots are more difficult to interpret than the 3D version. However, we agree that because of the color-scale used in the 3D version, the results regarding the z-parameter might have been confusing as previously displayed. Hence, for a better visualization of the z-parameter, we have changed the colorbar to the hsv format and we think now the variation of this parameter is more clear.

- lines 252 "in many empirical neurobiology datasets" - citations here?

Reply: Upon further inspection there is a substantial range of cross-validated prediction accuracies for brain-behavior associations. The range of performance appears largely determined by modality of method (e.g., multicellular arrays, BOLD imaging) as well as experimental setup. Rather than get lost in the weeds of the distinction, we opted to simply cut this sentence.

- lines 284-280 "grouped in two ways" and "the second CCA model". Took me a while to figure out that the "second CCA model" was referring to R->P, and not to the second way of grouping the firing rates. Perhaps disambiguate.

Reply: We have revised the text accordingly.

- Figure 7: define the colour code.

Reply: We have updated the caption of Figure 7 to do so.

- Methods, Table 1: why are GPe and STN an order of magnitude bigger than the other structures in the model, when they are orders of magnitude smaller?

Reply: We have added the following new text (lines 591-598) to address this point, “The relative population sizes were taken from past work [20,28]. The model includes more GPe neurons than SPN neurons, despite the relatively large size of the striatum, to allow for the divergence of connections from striatum to GPe [17,60], and we use the same number of STN neurons as GPe neurons to reflect the low divergence of connections from GPe to STN [61]. These larger numbers of GPe and STN neurons in the model also allow us to implement the relatively low connection probabilities between these regions [20,62-66].”

- Methods, Table 2: if this is a primate model, explicitly linked to human performance in the Results, what's the rationale for data taken from rats here?

Reply: While there do appear to be some key differences between species in terms of connectivity and complexity CBGT pathways, the critical properties (e.g., firing rates) of the cellular populations for our model appear to be largely preserved across species. The fact that we see similar ranges in rodents and primates gives further credence to the biological justification of our model. In addition, it provides a degree of conceptual flexibility for inferring cellular properties of certain populations in primates when no empirical data is present from primates (but exists for rodents).

- Methods, Table 3: STN-GPi, Th-Cx, and Th-CxI only have NMDA receptors listed, when they all have AMPA too as far as I know. Part of the model, or just an omission of a detail?

Reply: The table accurately reflects what is currently in the model. Our initial model simulations on AMPA showed a fairly weak influence on post-synaptic behavior (relative to NMDA) and hence we eliminated the AMPA receptors in these pathways to simplify the model. Heuristically, this weak effect makes sense: the relatively high and steady firing rates of the STN and thalamic populations result in a sustained excitatory signal transmitted to postsynaptic targets via the NMDA receptors, and rapidly decaying AMPA spikes on top of the sustained envelope generally have a small impact. While we do not think that this omission significantly influences our results, for future model simulations we will add AMPA receptors to these connections and explore their role more thoroughly.

- Methods, DDM model fitting: what noise level (sigma) was used for the fits?

Reply: We consider the default settings of the HDDM toolbox for determining the level of noise, which is sigma=1. We state this value in line 701 of the revised text.

- Methods, CCA: how was R^2 computed? (a) what are y_true and y_pred for CCA - I'm unclear on how the comparison is being made between "true" data and predicted: initially I thought these respectively the canonical

variates (i.e. u'X and u'Y) computed on the test data? (b) how was it computed over multiple components? As an average over the components, or by concatenating the predicted and true vectors, or...?

Reply: We changed the text to clarify this calculation (Section 4.4.1, lines 736-739) and added a relevant citation.
